# Basal Breast Cancer: A Complex and Deadly Molecular Subtype

**DOI:** 10.2174/156652412798376134

**Published:** 2012-01

**Authors:** F Bertucci, P Finetti, D Birnbaum

**Affiliations:** 1 Département d'Oncologie Médicale, Institut Paoli-Calmettes (IPC), Centre de Recherche en Cancérologie de Marseille, UMR891 Inserm, Marseille, France; 2 Département d'Oncologie Moléculaire, Institut Paoli-Calmettes, Centre de Recherche en Cancérologie de Marseille, UMR891 Inserm, Marseille, France; 3 Université Aix Marseille, Marseille, France

**Keywords:** Basal breast cancer, DNA microarrays, prognosis, triple-negative.

## Abstract

During the last decade, gene expression profiling of breast cancer has revealed the existence of five molecular subtypes and allowed the establishment of a new classification. The basal subtype, which represents 15-25% of cases, is characterized by an expression profile similar to that of myoepithelial normal mammary cells. Basal tumors are frequently assimilated to triple-negative (TN) breast cancers. They display epidemiological and clinico-pathological features distinct from other subtypes. Their pattern of relapse is characterized by frequent and early relapses and visceral locations. Despite a relative sensitivity to chemotherapy, the prognosis is poor. Recent characterization of their molecular features, such as the dysfunction of the BRCA1 pathway or the frequent expression of EGFR, provides opportunities for optimizing the systemic treatment. Several clinical trials dedicated to basal or TN tumors are testing cytotoxic agents and/or molecularly targeted therapies. This review summarizes the current state of knowledge of this aggressive and hard-to-treat subtype of breast cancer.

## INTRODUCTION

Despite recent advances in screening and treatment, breast cancer remains the most deadly cancer in women worldwide. During follow-up, up to 25% of patients experience a metastatic relapse from which they will succumb. Until recently, breast cancer was considered as a single disease with variable phenotype and expression of hormone receptors (HR; estrogen receptor, ER, and progesterone receptor, PR) and ERBB2 tyrosine kinase receptor. But breast cancer is a very heterogeneous disease and recent insights in our understanding of the disease were provided by genomics. Over the past decade, DNA microarrays [1] allowed genome-wide RNA expression profiling of breast cancer samples [2, 3], providing the unprecedented opportunity to tackle the complexity of the disease, and thus to improve the prognostic classification by identifying more homogeneous entities. In 2000, five molecular subtypes of breast cancer were recognized based on the gene expression patterns [4, 5]. The robustness and universality of this new taxonomy and its histoclinical correlations were then confirmed in different clinical forms of breast cancer and different ethnic populations [6]. Today, breast cancer is regarded as a collection of separate diseases, and subtyping is regarded as essential to better identify new molecular prognostic, predictive and/or therapeutic targets, an important step toward tailoring the treatment.

Among the subtypes, the basal subtype is particularly challenging. Basal tumors represent around 15% of invasive ductal breast cancers. They display distinctive epidemiological, phenotypic and molecular features with distinctive patterns of relapse, and a poor prognosis despite a relative chemosensitivity. Despite their relative scarcity, basal tumors cause a disproportionate mortality among breast cancer patients. In contrast to ER-positive and ERBB2-positive tumors, no targeted therapy is currently available for these tumors. This review describes our present knowledge of basal breast cancers and potential research directions, notably at the therapeutic level. 

## 1 DEFINITION OF THE BASAL SUBTYPE

**Fig. (1). Whole-genome clustering and molecular subtypes.**
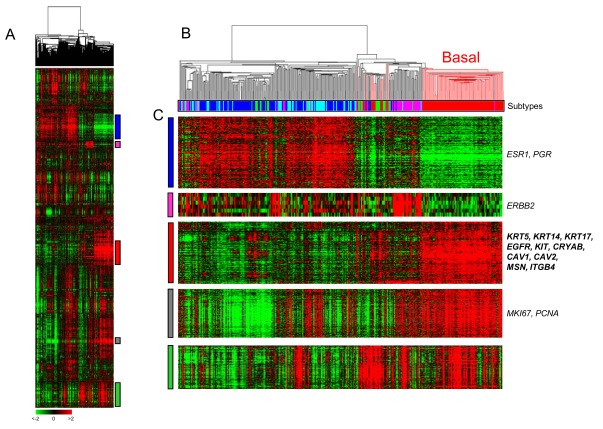
**A**/ Hierarchical clustering of 353 breast cancer samples profiled in our institution with 12.304 genes. Each row is a gene and each column a sample. The expression level of each gene in each sample is relative to its median abundance across the samples and is depicted according to the color scale shown under the matrix. Red and green indicate expression levels respectively above and below the median. Above the matrix, the dendrogram shows the degree of similarity between samples. To the right, vertical colored bars indicate gene clusters zoomed in C. **B**/ Dendrogram of samples. The branches are color-coded according to the molecular subtype: red for basal and black for the other subtypes. Under the dendrogram, the subtypes are color-coded as follows: dark blue, lulinal A; light blue, luminal B; pink, ERBB2; red, basal; green, normal-like. The basal subtype is the most homogeneous subtype. **C**/ Gene clusters of interest: luminal/ER-related, ERBB2, basal, proliferation and immune clusters. Some genes of interest of four clusters are noted (EntrezGene symbol). (For interpretation of the references to color in this figure legend, the reader is referred to the web version of this paper).

The first definition of basal breast cancer came from genomics and the Perou’s publication in 2000 [4]. Using DNA microarrays, the authors profiled 78 tumor samples from 42 patients, most of them treated with primary chemotherapy. For 20 patients, the pre- and post-chemotherapy samples were analyzed, allowing the definition of an “intrinsic” 500-gene set that accounted for most of the differences between patients. Clustering based on the expression of these genes revealed five major subtypes, which were biologically and clinically relevant (Fig. **1**). They were associated not only with the two principal normal epithelial mammary cell types (luminal and myoepithelial/basal) and with the two major molecular alterations of breast cancer (ER and ERBB2), but also with different clinical outcome. This new taxonomy confirmed the importance of hormone receptors and ERBB2, and provided new insights in the biology of disease. Two subtypes of predominantly ER-positive tumors (luminal A and B) - named luminal because of similarity of expression profiles with those of luminal mammary epithelial cells - were identified and associated with different survival. Similarly, three subtypes of predominantly ER-negative tumors were identified: basal, ERBB2 and normal-like. The basal tumors expressed genes associated with normal myoepithelial cells of the outer layer of duct breast, such as high molecular weight cytokeratins (CK5, CK14, CK17), along with smooth muscle markers, P-cadherin, caveolin 1, CD10, β4 integrin. By contrast, they did not express *ESR1*, *PGR* and *ERBB2*. This novel classification and its histoclinical correlations were then reproduced in larger series, on different platforms and by using different sample predictors by the same group [5, 7-11], and others in early [12-14], inflammatory [15, 16], and *in situ* breast cancers [17, 18], suggesting their robustness and universality. In most studies, the basal subtype was the most homogeneous of all subtypes in transcriptional term, even when three successively-published predictors [5, 9, 19] were applied [20].

**Fig. (2). Overlap between basal breast cancers and TN tumors.**
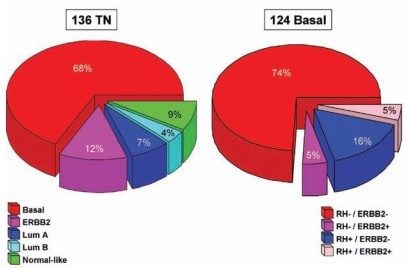
**A**/ Distribution of molecular subtypes within TN tumors. **B**/ Distribution of IHC groups (based on HR and ERBB2) within basal tumors. Our database was combined with publicly available MDA data [183]. (For interpretation of the references to color in this figure, the reader is referred to the web version of this paper).

However, because DNA microarrays are not routinely available in clinical practice, efforts were made to define basal breast cancer with standard pathological techniques such as immunohistochemistry (IHC), a simpler and more accessible assay. A wide variety of IHC surrogates have been proposed. Because most of basal tumors do not express RNA for *ESR1*, *PGR* and *ERBB2*, the triple-negative (TN: ER-/PR-/ERBB2-) definition, initially proposed, has been used widely. However, the overlap with the RNA-defined basal subtype is incomplete (Fig. **2**), with up to 30% discordance between the two definitions (RNA and IHC) [21-23]. The incomplete overlap between basal and TN breast cancers could translate true differences in their biology. Triple-negative tumors represent a more heterogeneous group than basal tumors, and include basal and non-basal tumors very different both at the histoclinical and molecular level notably for expression of potential therapeutic targets [22]. Several new subtypes of TN tumors were identified. The claudin-low subtype was characterized by a low expression of many claudin genes (notably 3, 4 and 7) involved in epithelial cell tight junctions [24-27]. Six subtypes were identified in a large dataset of 587 TN cases, including two subtypes resembling the basal subtype [28], Thus, to define a more homogeneous class of basal breast cancer while avoiding a definition based on negative staining, more sophisticated definitions have been proposed, which include positive staining for one or several basal/myoepithelial markers such as CK5, CK14, CK17, P-cadherin, KIT, EGFR and/or others. The most frequently quoted one defined basal-like tumors as ER and ERBB2-negative, CK5/6 and/or EGFR-positive [29]; this definition was recently modified by the addition of negative PR staining [30]. Other composite IHC basal-like definitions have been published (see [31] for a comprehensive list). 

Today, no consensus has been reached regarding the optimal definition of basal tumors, and although not completely synonymous, the RNA and IHC definitions are used interchangeably. If the goal is to define a homogeneous subtype of cancers due to specific molecular alterations and similarly sensitive to treatment, the RNA definition should be the gold standard. Compared to IHC, DNA microarrays are more quantitative, more prone to standardization and automatization, and associated with less pre-analytical and technical variability, less subjectivity, and higher reproducibility. The two major drawbacks of the RNA definition are the limitation of its application in clinical routine and the need for a better standardization. Reciprocally, two major drawbacks of the IHC definitions, beside the issue of standardization, are the potential for misclassification due to a less thorough definition, and for composite definition. Efforts are ongoing to develop assays able to define in routine practice the intrinsic molecular subtypes including the basal one. Ideally, it should combine the advantages of both DNA microarray technology and IHC. At least two assays that classify breast cancers into gene expression-based subtypes have been recently launched: Breast Bioclassifier (ARUP Laboratories, Salt Lake City, UT, USA), a 55-gene qRT-PCR assay that uses formalin-fixed, paraffin-embedded samples, and BluePrint (Agendia, Amsterdam, Netherlands), a 80-gene DNA microarray assay that uses fresh samples fixed in an RNA-protective solution. To date, the most frequently used definition is the genomic one in research studies, and the IHC one in clinical trials. Because of this incomplete overlap it is important to precise the definition that is used in all reports. Hereafter, the term “basal” will refer to the genomic definition, “TN” to the triple-negative status, and “basal-like” to the IHC definition (4 or 5 protein markers). 

## 2 MOLECULAR ASPECTS

Basal tumors express low RNA levels of *ESR1*, *PGR* and *ERBB2*, and high levels of proliferation genes (Fig. **1**). They also specifically overexpress a “basal” gene cluster. The high expression of some “basal” genes was confirmed at the protein level. Examples include P-cadherin [32], KIT [29], EGFR [33], MET [34], caveolin 1 and 2 [35], β4-integrin [36], α-basic crystalline [37], and moesin [38]. Some of these proteins (EGFR, P-cadherin, α-basic crystalline, and moesin) are independent poor-prognosis markers in breast cancer. Comparative analysis of whole-genome expression data of basal and luminal A samples showed a great extent of transcriptional differences between the two subtypes, with more than 5.500 of 30.000 probe sets found as differentially expressed [39]. Genes associated with signal transduction, angiogenesis, cell cycle and proliferation, cell survival, DNA replication and recombination, motility and invasion, and NFkB signaling are overexpressed in basal tumors. Interestingly, several of them code for therapeutic targets (see below). In a pooled analysis of 2,485 invasive breast cancer samples, the highest *PARP1* mRNA expression was observed in the basal subtype compared to the other subtypes, with strong association between mRNA expression and gene copy gain [40]. 

Loss of PTEN and activation of the PI3K/AKT pathway [41], and *TP53* mutations are frequent in basal breast cancers [5, 8]. ArrayCGH-based profiling [42-45] showed a high degree of genomic instability in the basal subtype (“complex pattern”) with frequent low-level gene copy number alterations (gains and losses), but less frequent high-level alterations (amplifications and deletions). Similarly, a high rate of loss of heterozygosity (LOH) was reported [46]. Regions altered in basal samples such as 6p21-p25, 12p13 (gained) or 5q11 (lost) likely harbor candidate oncogenes and tumor suppressor genes respectively, which remain to be identified. Inactivation of the RB pathway is also frequent and constitutes another reason of genome instability [47, 48]. However, not all basal breast tumors have a highly rearranged genome [49]. 

Sporadic basal breast cancers and hereditary *BRCA1*-associated breast cancers share several morphological, immunohistochemical and biological features including high proliferation, poor differentiation, high grade, triple negativity, TP53-positivity, expression of basal cytokeratins and markers [50] and cell-of-origin (see below). This community is reflected at the genomic and transcriptional levels with genome instability, similar patterns of X-chromosome inactivation [51], and presence of *BRCA1*-mutated tumors within the basal subtype [5]. Other resemblances lie in the clinical outcome with similar poor prognosis, and a similar pattern of metastatic relapse [52, 53]. All these similarities strongly suggest a fundamental defect in the BRCA1 DNA-repair pathway in sporadic basal breast cancers [54]. *BRCA1* is rarely mutated in sporadic mammary tumors overall, but more frequently in TN tumors [55, 56]. Other mechanisms of BRCA1 inactivation in basal tumors include *BRCA1* promoter methylation [57, 58], transcriptional inactivation due to the overexpression of *ID4* (negative regulator of *BRCA1* transcription) [54], and other mechanisms such as BARD1 inactivation [59]. Whether BRCA1 inactivation is a cause or a consequence of the basal phenotype is not clear. Two hypotheses have been formulated to explain these resemblances: i) better tolerance to loss of BRCA1 function in basal tumors, perhaps due to the inactivation of other tumor suppressor genes such as *TP53*, ii) absence of differentiation of epithelial cells due to loss of *BRCA1*, and absence of transition from ER-negative to ER-positive status, leading to tumors with a stem cell-like basal phenotype [60-62]. The inactivation of BRCA1, involved in repair of double-strand DNA breaks, partially explains the genomic instability of basal breast cancers, and theorically confers sensitivity to chemotherapy agents causing inter-strand and double-strand breaks [63] and to PARP inhibitors (see below).

In addition to these distinctive molecular features of cancer cells themselves, basal breast cancers also present distinctive microenvironment and stromal-epithelial interactions [64]. Comparative co-cultures of basal and luminal breast cancer cell lines with fibroblasts showed differential expression of numerous interleukines and chemokines (including IL-6, IL-8, CXCL1, CXCL3, and TGFβ) by basal cell lines and increased migration *in vitro* in basal tumors. These phenotypes and gene expression changes invoked by cancer cell interactions with fibroblasts support the microenvironment and cell-cell interactions as intrinsic features of breast cancer subtypes.

## 3 CELL-OF-ORIGIN OF THE MOLECULAR SUBTYPES

The epithelium of the mammary gland has two layers of cells. The inner, luminal layer lines the lumen of the breast duct and lobule. Luminal cells express the ER, low molecular weight cytokeratins (CK7, CK8, CK18 and CK19) and* PGR*, *GATA3*, *BCL2 *and other ER-induced genes. Luminal tumors express these genes. The outer layer of mammary epithelium is the myoepithelial layer. Myoepithelial cells express CK5/6, CK14, alpha-smooth actin, P-cadherin and CD10. Adjacent to the basement membrane, they are sometimes confusingly called basal cells. Other basal cells expressing basal cytokeratins CK5/6 and CK14 are interspersed in the two layers. These basal cells are thought to be immature progenitors and stem cells. Breast cancers that express basal cytokeratins have been called basal but their cell-of-origin is not known [65, 66]. 

It has been suggested that the different subtypes of breast cancer originate from mammary stem or progenitor cells at different stages of lineage differentiation [67]. Mammary stem cells express several genes in common with basal breast cancers [68]. Reciprocally, basal breast cancers express stem cell genes [69, 70]. These similarities do not necessarily imply derivation, but provide a working hypothesis. A basal breast cancer probably derives from a stem or progenitor cell that has never expressed ER. *BRCA1* breast cancers derive from an ER-negative luminal progenitor [71, 72]. BRCA1 may be required for the transition from an ER-negative to an ER-positive progenitor [73]. Loss of BRCA1 function in basal breast cancer is in agreement with this finding. In contrast, luminal cancers may derive from an ER-positive luminal progenitor [74]; due to specific alterations [75], in luminal B breast cancers this progenitor may have lost ER expression. A basal tumor may represent cells arrested at an early stage of differentiation and devoid of differentiation markers and hormone receptors. The extent of difference in gene expression we have evidenced between basal and luminal breast cancers [39] is compatible with this possibility. No doubt that the elucidation of the cellular hierarchy in both normal human mammary gland and in the different breast cancer subtypes will improve our understanding of breast cancer. 

## 4 EPIDEMIOLOGICAL ASPECTS AND PRECURSOR LESIONS

Basal breast cancers represent 15-25% of breast cancers, whatever the definition used. Significant interactions of the basal subtype with age and race have been evidenced (Table **1**). The average age of patients with basal invasive breast cancer is or tends to be younger than the age of other patients [39, 76-78]. Large population-based studies have reported a higher frequency of basal breast cancers among premenopausal women [8, 79-81]. Higher incidence is also found in African American women when compared with non-African American women [8, 79, 81-83]. For example, the respective frequency of basal tumors is 26% *versus* 16% in the Carolina Breast Cancer Study (CBCS) [8], and 21% *versus* 10% in the SEER (Surveillance Epidemiology and End Results) database [81]. In the CBCS, the patients at highest risk to have a basal breast cancer are premenopausal African American women, in whom they represent 27 to 47% of cases [8]. 

Reanalysis of classical risk factors for breast cancer in two large population-based studies revealed differences according to the IHC-defined subtypes. In the CBCS [8, 84], the risk factors associated with basal tumors, but not with luminal A tumors, included a younger age (inferior to 26 years) at first full-term pregnancy, higher parity, absence of or shorter duration of breast-feeding, lower number of breast-fed children, younger age at menarche, the use of medications to suppress lactation and higher body mass index (BMI). The younger age at menarche and the high BMI were confirmed in the Polish Breast Cancer Study [77]. The existence of various, distinct and sometimes opposite risk factors between the subtypes, notably basal and luminal A, further suggests etiologic heterogeneity of breast cancer, and call for subtype-specific epidemiological studies and approaches of prevention. 

At the molecular level, the presence of a *BRCA1* mutation strongly increases the risk to develop a basal breast cancer. Genome-wide association studies in unselected populations have reported other associations with genetic loci [85, 86], with several links found in ER-positive tumors [87, 88]. A significant association between the G/G genotype (combination of G and G alleles at the locus) of a non-synonymous *MYBL2* germline variant and an increased risk of basal breast cancer was recently reported [89]. Further studies in subtype-specific series are awaited.

Regarding the precursor lesions of invasive basal breast cancers, several studies have demonstrated that a subset of *in situ* ductal carcinomas (DCIS) are basal as defined by using a genomic [17, 18] or an IHC definition [90-94]. In most cases, basal DCIS were associated with unfavorable prognostic variables such as high nuclear grade, presence of necrosis, high proliferative index and p53 overexpression. In the CBCS, the prevalence of basal DCIS was 8% [92], inferior to that observed for invasive basal tumors (20%) [8]. Interestingly, the age of patients with basal breast cancer at diagnosis was similar to that of patients with another cancer subtype for *in situ* tumors [92], whereas it was lower in case of invasive tumors [8]. These two discrepancies (prevalence and age) likely reflect the more rapid progression rate of basal breast cancers. The identification of basal DCIS intimately admixed with invasive basal breast cancers suggests that basal DCIS could serve as precursor lesions for invasive cases [92]. Earlier precursor lesions, such as atypical ductal hyperplasia, for basal DCIS remain to be identified.

## 5 HISTOCLINICAL ASPECTS

Data are rather consistent in the histoclinical characteristics of the basal subtype, regardless of the definition used (Table **1**). Most basal tumors are invasive ductal cancers, but occasionally may be typical or atypical medullary [95, 96], metaplastic, adenoid cystic, squamous-cell, or mucoepidermoid [97]. Classically, they are high-grade tumors, with more than 75% being grade III [8, 39, 76, 98]. They display a high mitotic index – which likely explains their overrepresentation among the cancers diagnosed between annual mammograms (“interval cancers”) [99] – as well as high nuclear/cytoplasmic ratio, pushing margins of invasion, central necrosis, lymphocyte-rich stroma, and frequent apoptotic cells [100]. 

Results are more conflicting regarding the correlation of basal subtype with the pathological tumor size as compared to other subtypes: some studies identified correlation with higher size [9, 76, 98] whereas others did not find any correlation [80, 101, 102]. In a pooled series of 480 luminal A cases and 285 basal cases defined upon the intrinsic gene set [39], we observed a higher size for basal tumors at a discriminatory threshold of 2 cm. Data also vary regarding the pathological axillary lymph node status, with either lower rates of positivity as compared with other subtypes [39, 76, 102], or similar rates [8, 80]. Interestingly, correlation between pathological tumor size and axillary lymph node status is absent [39] or weak [103] in basal tumors, whereas it is present in luminal A tumors. This uncoupling of size and node involvement in basal tumors, combined with their high metastatic risk, might reflect a preferentially hematogeneous metastatic spread and/or an underlying disproportionate relationship between the number of cancer cells with lymph metastatic potential and the size of the cancer.

## 6 THERAPEUTIC RESPONSE

The frequent ER-negativity of basal breast cancers as well as their high grade with high proliferative index [104] should theoretically confer them sensitivity to chemotherapy, notably to drugs classically used in breast cancer. This was confirmed by most neo-adjuvant anthracycline and/or taxane-based chemotherapy studies, which documented a higher rate of pathological complete response (pCR) in the basal subtype than in any other subtype [14, 15, 105]. In a small series of 21 inflammatory breast cancers (IBC), we reported a pCR rate of 80% in the basal subtype and 27% in the luminal A subtype after anthracycline-based chemotherapy [15]. In a series of 100 non-IBCs treated with paclitaxel followed by anthracycline-based regimen, pCR rate was 45% in basal tumors and in ERBB2+ tumors, but only 6% in luminal tumors and 0% in normal-like tumors [14]. However, the basal subtype did not remain an independent predictor of pCR after adjustment for other histoclinical features. Higher response rates were reported for TN breast cancers compared to non-TN cases [106-109]. Finally, in a pooled analysis of eight German neo-adjuvant trials, patients with TN breast cancer benefited more than the other patients from dose-intense chemotherapy [110]. However, despite this relatively high rate of pCR, basal tumors are associated with a relatively poor prognosis: this is the “triple-negative paradox” [105]. In fact, the prognosis is similarly good for patients with pCR regardless of subtype, but is worse in TN cancers as compared with non-TN cancers in those patients in whom pCR is not achieved [105, 108]. 

This higher relapse rate among patients with basal breast cancer calls for the development of more effective first-line chemotherapy regimens, all the more so that these patients who usually relapse shortly after (neo)adjuvant chemotherapy should be considered as resistant to anthracyclines and taxanes. In the metastatic setting, the notions of disease aggressiveness, relatively young age, visceral locations, and TN status call more for the use of combination chemotherapy than single-agent sequential chemotherapy. In the case where the tumor is resistant to anthracycline and taxane, other available drugs include capecitabine, vinorelbine, Nab-paclitaxel, ixabepilone, and gemcitabine. However, it remains unclear whether one of them is more efficient as single-agent in basal/TN breast cancers, and today there is no regimen specifically recommended for metastatic TN patients. The promising effect of platinum salts according to BRCA-deficiency is described in the last section dedicated to therapeutic perspectives.

In the adjuvant setting, some groups have addressed the benefit of different regimens of chemotherapy according to the subtypes. Data come from large retrospective series of samples deposited onto tissue microarrays and analyzed using IHC. In this setting, present data are more complex to interpret than in the neo-adjuvant setting. Most studies showed a benefit of adjuvant chemotherapy in TN or basal-like tumors [29, 30, 111-116]. They also suggested a relative benefit of non-anthracycline regimen (CMF: cyclophosphamide, methotrexate, 5-fluorouracil) [29, 30, 113, 114] and a relative anthracycline resistance [101, 111], a benefit of high-dose regimens [112, 115, 117, 118], and a benefit of paclitaxel [116] or docetaxel [119, 120] addition to anthracyclines. To date, these data remain too preliminary to draw any conclusion. They call for larger prospective studies to validate or not the predictive value – independent or not - of basal subtype for tumor chemosensitivity, and to define the optimal regimen.

## 7 PATTERN OF RELAPSE AND SURVIVAL 

The prognosis of basal subtype is poorer than that of other subtypes (Table **1**). Most of gene profiling studies have repeatedly reported a shorter metastasis-free survival (MFS) and overall survival (OS) among basal breast cancer patients [5, 7, 9, 12, 15, 39, 80, 121, 122]. According to three different multigene expression signatures (70-gene signature, recurrence score and wound response signature) most of the tumors predicted as poor-prognosis were basal [123]. In our pooled series of 480 luminal A and 227 basal breast cancers, the 5-year OS was 88% for patients with luminal A subtype and 58% for patients with basal subtype, and the 5-year MFS was of 82% and 66%, respectively [39]. Data are less consistent with the IHC definitions. Most studies [8, 29, 98, 101, 108, 113, 114, 124-127] showed that the clinical outcome of TN breast cancers is less favorable than that of non-TN cancers. However, some studies did not find such association [76, 102, 128]. This discrepancy of outcome for basal subtype between the gene and IHC definitions is well evidenced by our study in which the basal subtype was defined using the intrinsic gene set [39]. No difference for MFS existed among the 160 basal tumors between those with and those without the TN status. Conversely, there was a significant difference between 123 TN samples defined as basal (shorter MFS) and 49 TN samples defined as non-basal. This observation was confirmed using an IHC definition of basal (positivity of EGFR and/or CK5/6) within a series of TN samples [129]. A confrontation of two IHC definitions of basal breast cancers in a series of 3744 cases [30] revealed that the five-biomarker definition (ER, PR, ERBB2, CK5/6 and EGFR) had superior prognostic value than the TN one.

**Fig. (3). Survival according to molecular subtypes.**
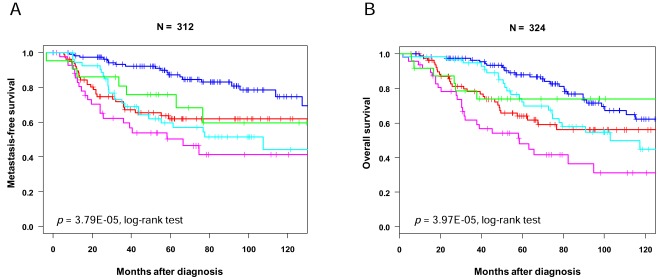
Kaplan-Meier curves for metastasis-free survival (**A**) and overall survival (**B**) according to subtypes in our series of 353 patients treated in our institution. The color legend is similar to Fig. (**2**). (For interpretation of the references to color in this figure, the reader is referred to the web version of this paper).

Basal breast cancers have a pattern of metastatic relapse distinct from the luminal cancers. Regarding the timing, they are more likely to metastasize during the first 3 years of follow-up (Fig. **3**). The risk of recurrence declines thereafter, conversely to luminal A cancers that display a more consistent rate over the follow-up [8, 39, 108]. This observation explains the absence of difference in survival reported by some studies between basal and luminal tumors after a 10-years follow-up [121, 125]. Regarding the location of metastases, basal breast cancers develop visceral metastases, notably brain and lung, more frequently than the luminal cancers, but develop less frequently bone and axillary lymph node metastases [102, 108, 130-133]. In a series of 3000 breast cancer patients with brain metastasis [134], the TN status was the strongest risk factor for brain relapse. These observations and the difference in pathological tumor size / lymph node status correlation between the basal and luminal subtypes suggest different routes for metastasis. 

Although the discrepancies reported across studies regarding the prognosis and the response to chemotherapy may reflect differences in treatments and populations, they may also reflect the heterogeneity of basal breast cancers. Not all patients have an unfavorable clinical outcome. To date, reliable identification of basal breast cancer patients with a good or a poor prognosis is difficult and based only using histoclinical features, which are far from being optimal [135-137]. But these reported prognostic studies have so far concerned basal tumors defined using the TN definition only. We [39] and others [30, 129] showed that the basal subtype was associated with poor survival within TN cancer women. A pooled analysis [138] showed that seven tested prognostic multigene expression signatures [69, 139-145] performed very well in the ER+/ERBB2- subgroup (probably because they all measure proliferation, a major factor of prognosis in this population), but were not at all informative for the TN subgroup. In this subgroup, the major prognostic factor was an immune response module, the expression of which is associated with better survival. Similar results were observed in the rare studies dedicated to ER- tumors [146-150], which in fact, for three of them, included basal and ERBB2+ tumors. In two studies dedicated to basal tumors only, we confirmed the favorable prognostic impact of activation of cytotoxic tumor-infiltrative lymphocytes [151, 152]. 

Few data exist regarding the association of basal subtype with the rate of loco-regional recurrence. Some groups have reported the absence of differences with the other subtypes (IHC definition) [98, 153]. But many others have shown an increased risk of local and/or regional recurrences after breast-conserving therapy [114, 127, 154, 155], and after mastectomy with and without radiation therapy suggesting that TN breast cancers do not benefit form radiation therapy after mastectomy [156]. 

## 8 SYSTEMIC TREATMENTS: PERSPECTIVES

**Fig. (4). Therapeutic strategies under assessment in basal and/or TN breast cancer.**
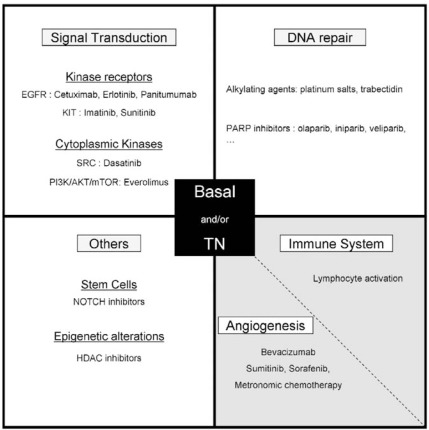
White: tumor cells; light grey: tumor microenvironment.

The frequent triple-negativity of basal breast cancers does not render them candidate to hormone therapy and anti-ERBB2 therapies, and until now, chemotherapy represented the sole available systemic treatment. However, the recent insights in the pathogenesis of these tumors are being translated into the development of new therapeutic strategies targeting molecular alterations (Fig. **4**). Clinical trials are underway, which undoubtedly, will contribute to enlarge our therapeutic armamentarium in a near future. We present here some promising research directions (for more exhaustive reviews, see [157, 158]).

The first strategies exploit the defect in double-strand DNA break repair mechanisms. Regarding chemotherapy, this defect should confer sensitivity to certain drugs [63, 159], notably the DNA-damaging agents like platinum compounds [160], mitomycin-C [161], anthracyclines, etoposide and bleomycin. To date, a few clinical data, if any, support these *in vitro* observations. For platinum salts, two neo-adjuvant trials of single-agent cisplatinum reported high pCR rates: 90% in a series of 10 *BRCA1*-mutated TN patients [162], and 22% in a series of 28 TN patients unselected for the *BRCA* mutation status [163], further reinforcing the hypothesis that, among the TN patients, those with a *BRCA1*-deficient tumor such as basal tumors, are highly sensitive to platinum [28]. That was confirmed in a retrospective study, which revealed that such tumors are more sensitive to platinum compounds than to non-platinum-based regimens [164]. In the pre-treated metastatic setting, two trials reported clinical response rates of 17% and 30% after respectively carboplatin plus cetuximab [165] and carboplatin plus irinotecan [166]. Larger and comparative series are required, and several clinical trials are ongoing with platinum salts, such as the CALGB 40603 trial, a 2 x 2 randomized neo-adjuvant trial that plans to enrol 362 TN patients (NCT00861705). Another promising alkylating agent is trabectedin [167]. Several other drugs, such as taxanes, gemcitabine, and metronomic chemotherapy, are under evaluation.

The other way to exploit the DNA repair defect is the use of poly (ADP-ribose) polymerase (PARP1) inhibitors. This enzyme is critical in the base excision repair of single-strand DNA breaks. In its absence, single-strand breaks degenerate to double-strand breaks, which are not repaired if BRCA1 is deficient [168]. Several PARP1 inhibitors (iniparib, olaparib, and veliparib), alone (as agent causing synthetic lethality) or in combination with chemotherapy (as chemopotentiating agent), are in clinical development in patients with TN or *BRCA1*-associated breast cancers. Promising results were initially reported with iniparib (BSI-201) and olaparib. A phase II study of oral olaparib in pretreated metastatic *BRCA*-mutated patients (57% were TN) showed 41% response rate with the 400-mg dose [169]. In a phase II trial of 123 metastatic TN patients [170], a combination of intravenous iniparib and chemotherapy (carboplatin-gemcitabine) improved the rates of response (from 16 to 48%) and of clinical benefit (21 to 62%), as well as the progression-free survival (PFS: median: 3.6 months to 5.9) and overall survival (median: 7.7 months to 12.3) compared with chemotherapy alone. However, these results did not hold up in the following phase III trial that enrolled 519 TN patents pretreated with two or fewer metastatic regimens [171]. All patients received gemcitabine and carboplatin and were randomized to iniparib or placebo. The one-month improvement in PFS (median: 4.1 to 5.1 months) with iniparib and the increase of less than one month in OS (median: 11.1 to 11.8 months) did not meet the prespecified definition of statistical significance. An exploratory analysis suggested that patients who received iniparib as second- or third-line therapy might have benefited from treatment, Another PARP inhibitor, veliparib, given in combination with temozolomide in a phase II trial of metastatic breast cancer patients showed that responses were limited to BRCA-associated cases [172], further suggesting the need for proper patient selection. Resistance to PARP inhibitors has been observed *in vitro* due to the restoration of a functional BRCA2 isoform resulting from a gene deletion [173]. 

Anti-angiogenic agents are under evaluation in TN breast cancers. In the ECOG 2100 trial, which compared weekly paclitaxel with and without bevacizumab, a monoclonal antibody directed against VEGF, TN patients benefited from bevacizumab as much as the average [174]. In a neo-adjuvant phase II study, bevacizumab associated with cisplatinum led to 37% pathological responses [175]. In a phase II study, the multikinase VEGFR inhibitor, sunitinib, given as single agent in anthracycline and taxane-pretreated metastatic patients, yielded a 15% response rate in the TN subgroup, slightly higher than the 11% rate observed in the whole population [176]. Two studies assessing sorefenib in the metastatic setting gave discordant results regarding the benefit in the TN subgroup [177, 178]. To date, it remains unclear whether TN/basal breast cancers are more sensitive than others to anti-angiogenic drugs. Bevacizumab is being tested in the neo-adjuvant CALGB 40603 trial, which includes a second randomization (with *vs*. without the drug) in each chemotherapy arm. 

Several other potential targets for TN tumors are involved in signal transduction pathways. EGFR is frequently overexpressed in basal breast cancers [29], and EGFR inhibitors are under evaluation. In the completed TBCRC 001 study, cetuximab, a monoclonal antibody directed against EGFR, was given alone and in association with carboplatin in pretreated TN metastatic patients [165]. The response rate was modest (17%) with the combination; it was even lower (6%) with cetuximab alone, but suggested some activity in selected patients. Interestingly, when serial tumor biopsies could be done, a perfect correlation was observed between the clinical benefit and the demonstration of an EGFR pathway deactivation (observed in 25% of cases). Another completed phase II trial compared irinotecan plus carboplatin with *versus* without cetuximab [166]: the response rate was higher with the antibody (49 *vs*. 30%), but the PFS was similar. The high failure rate with EGFR inhibitors relatively to the frequent overexpression may be due to the absence of pathway activation (*EGFR* gene amplification is rare) or the existence of alternative activation pathways such as the frequently observed PTEN inactivation and AKT activation in TN breast cancers. Several other EGFR inhibitors are being assessed in TN patients such as erlotinib and panitumumab. 

Other inhibitors of signal transduction under development target second messengers. Examples include everolimus, a mammalian Target Of Rapamycine (mTOR) inhibitor, and dasatinib, which inhibits ABL and SRC family kinases. Indeed, the frequent mTOR activation observed in TN breast cancers, and the fact that mTOR activation has been associated with cisplatinum resistance, which can be overcome with mTOR inhibitors [179], argue for the ongoing development of everolimus in TN breast cancer, alone and in combination with cisplatinum-based regimen. Regarding dasatinib, pre-clinical data have shown that basal breast cancer cell lines are particularly sensitive to this inhibitor [180, 181]. In a phase II trial of single-agent dasatinib in pretreated metastatic TN patients, the response rate was low (4.7%) with a median PFS of 8.3 weeks [182].

Other examples of potential therapeutic targets overexpressed in basal breast cancers include the NFkB pathway, the tyrosine kinase receptor MET, or the chemokine receptor CXCR4 and its ligand CXCL12/SDF1. Finally, the favorable prognostic impact of the lymphocyte activation in basal breast cancer and the identification of new antigens suggest that strategies aimed at stimulating the immune system should be tested. Identification of protein networks and pathways that control breast cancer stem cells should also help design new drugs. 

Most of trials are ongoing, and many others will be soon activated in the metastatic, neo-adjuvant and also adjuvant settings. Given the results of the first completed trials, caution is required for the interpretation of the results and the selection of patients in future trials for at least two reasons. First, initial studies were not directed specifically at TN breast cancers but at all breast cancers, arising the issue of unplanned subset analyses that often do not have the statistical power to detect significant differences. Second, the inclusion criteria of theorical basal tumors, which use the imperfect TN definition, led in fact to the enrollment of basal and non-basal tumors very different at the histoclinical level, but also for the RNA expression of the theorical therapeutic target [22]. Ideally, the development of a companion molecular test for better selecting the patients should be associated to better understand the impact of the drug. In this context a retrospective evaluation of basal markers and the search for companion markers will have to be done, notably in the negative or non-significant trials to attempt to document a positive impact in the basal population or the marker-positive subset, provided that tissue samples have been collected prospectively. 

## CONCLUSION

Genomics has modified our view of breast cancer, which is currently considered as a group of molecularly distinct diseases. The basal subtype represents a challenging subtype with distinctive epidemiological, histoclinical, and molecular features, with distinctive patterns of relapse, poor prognosis despite relative chemosensitivity, and no available targeted therapy. Currently, no routine diagnostic procedure exists specifically for this subtype, and the patients’ management is similar to that of other subtypes regarding prevention, prognostic assessment and treatment. A detailed molecular characterization of basal tumors is ongoing, both to better understand their different biology and clinical outcome, and to identify specific diagnostic, prognostic, and therapeutic targets. Today, no cytotoxic or targeted agent has yet been registered specifically in TN or basal breast cancer patients, but several targeted drugs are under development, which might improve the patients’ survival.

## Figures and Tables

**Table 1.** Characteristics of Basal/TN Breast Cancers

**Epidemiological features**	Younger age
	Pre-menopausal status
	African-American race
	High BMI
	Younger age at menarche
**Histoclinical features**	Ductal carcinoma (and medullary)
	High-grade
	High mitotic index
	Nuclear pleomorphism
	Pushing margins of invasion
	Central necrosis
	Negative ER, PR and ERBB2 IHC staining
	Poor correlation between pathological tumor size and axillary lymph node status
**Molecular features**	*TP53* mutations
	BRCA1-deficiency
	RB inactivation
	Genome instability (« complex pattern »)
**Prognosis **	Poor prognosis
	Early relapses (first 3 years)
	Visceral metastases (brain, lung)
**Therapeutic response**	Sensitive to primary chemotherapy
	No validated targeted therapy (ongoing trials)

## ABBREVIATIONS

ArrayCGH: = array-based comparative genomic hybridization
BMI: = body mass index
CBCS: = Carolina Breast Cancer Study
CMF: = cyclophosphamide, methotrexate, 5-fluorouracil
DCIS: = ductal carcinoma *in situ*
ER: = estrogen receptor
HR: = hormone receptor
IBC: = inflammatory breast cancer
IHC: = immunohistochemistry
LOH: = loss of heterozygosity
MFS: = metastasis-free survival
OS: = overall survival
pCR: = pathological complete response
PFS: = progression-free survival
PR: = progesterone receptor
TN: = triple-negative
